# Supplementary material for: Molecular Remodeling of the Sperm Proteome Following Varicocele Sclero-Embolization: Implications for Semen Quality Improvement
Source: Proteomes. 2025 Jul 15;13(3):34. doi: 10.3390/proteomes13030034 (PMC12286009; doi:10.3390/proteomes13030034)
Supplement: Supplementary file 1 [file proteomes-13-00034-s001.zip › Suppl file S3 Diff.expressed POST3M_PRE.pdf]

DIFFERENTIAL PROTEIN EXPRESSION BASED ON FOLD CHANGE POST 3M / PRE

Increase of expression = Log2(Fold Change) > 1.5

Decrease of expression = Log2(Fold Change) < -1.5

163 Up-regulated

ID	FC	Log2FC	Description
P15309	4.673203	2.224412	Prostatic acid phosphatase OS=Homo sapiens OX=9606 GN=ACP3 PE=1 SV=3
P08118	4.986849	2.318128	Beta-microseminoprotein OS=Homo sapiens OX=9606 GN=MSMB PE=1 SV=1
P07288	6.260489	2.646275	Prostate-specific antigen OS=Homo sapiens OX=9606 GN=KLK3 PE=1 SV=2
Q5JQC9	2.829453	1.500523	A-kinase anchor protein 4 OS=Homo sapiens OX=9606 GN=AKAP4 PE=1 SV=1
P49221	3.286573	1.716584	Protein-glutamine gamma-glutamyltransferase 4 OS=Homo sapiens OX=9606 GN=TGM4 PE=1 SV=2
P25311	6.118383	2.61315	Zinc-alpha-2-glycoprotein OS=Homo sapiens OX=9606 GN=AZGP1 PE=1 SV=2
Q9UIA9	4.092877	2.033115	Exportin-7 OS=Homo sapiens OX=9606 GN=XPO7 PE=1 SV=3
P61769	2.926866	1.549357	Beta-2-microglobulin OS=Homo sapiens OX=9606 GN=B2M PE=1 SV=1
P09466	3.719576	1.895138	Glycodelin OS=Homo sapiens OX=9606 GN=PAEP PE=1 SV=2
P07686	2.994519	1.582324	Beta-hexosaminidase subunit beta OS=Homo sapiens OX=9606 GN=HEXB PE=1 SV=4
Q6W4X9	4.104308	2.037139	Mucin-6 OS=Homo sapiens OX=9606 GN=MUC6 PE=1 SV=3
Q14508	3.373725	1.754342	WAP four-disulfide core domain protein 2 OS=Homo sapiens OX=9606 GN=WFDC2 PE=1 SV=2
P01034	3.461265	1.791299	Cystatin-C OS=Homo sapiens OX=9606 GN=CST3 PE=1 SV=1
P05154	4.294431	2.102467	Plasma serine protease inhibitor OS=Homo sapiens OX=9606 GN=SERPINA5 PE=1 SV=3
P20151	3.278273	1.712936	Kallikrein-2 OS=Homo sapiens OX=9606 GN=KLK2 PE=1 SV=1
P35527	24.83409	4.63425	Keratin, type I cytoskeletal 9 OS=Homo sapiens OX=9606 GN=KRT9 PE=1 SV=3
P30153	2.873122	1.522619	Serine/threonine-protein phosphatase 2A 65 kDa regulatory subunit A alpha isoform OS=Homo sapiens OX=9606 GN=PPP2R1A PE=1 SV=4
Q96KP4	2.881431	1.526785	Cytosolic non-specific dipeptidase OS=Homo sapiens OX=9606 GN=CNDP2 PE=1 SV=2
Q8N807	3.93435	1.976125	Protein disulfide-isomerase-like protein of the testis OS=Homo sapiens OX=9606 GN=PDILT PE=1 SV=2
P13646	5.174995	2.371558	Keratin, type I cytoskeletal 13 OS=Homo sapiens OX=9606 GN=KRT13 PE=1 SV=4
P15144	2.842106	1.506961	Aminopeptidase N OS=Homo sapiens OX=9606 GN=ANPEP PE=1 SV=4
P01036	10.6597	3.414095	Cystatin-S OS=Homo sapiens OX=9606 GN=CST4 PE=1 SV=3
O14841	3.8636	1.949946	5-oxoprolinase OS=Homo sapiens OX=9606 GN=OPLAH PE=1 SV=3
P11766	3.07707	1.621557	Alcohol dehydrogenase class-3 OS=Homo sapiens OX=9606 GN=ADH5 PE=1 SV=4
Q04760	2.972977	1.571908	Lactoylglutathione lyase OS=Homo sapiens OX=9606 GN=GLO1 PE=1 SV=4
Q99985	3.290294	1.718216	Semaphorin-3C OS=Homo sapiens OX=9606 GN=SEMA3C PE=2 SV=2

P12429	3.953159	1.983006	Annexin A3 OS=Homo sapiens OX=9606 GN=ANXA3 PE=1 SV=3
Q04917	9.331233	3.222068	14-3-3 protein eta OS=Homo sapiens OX=9606 GN=YWHAH PE=1 SV=4
P02647	4.410472	2.140933	Apolipoprotein A-I OS=Homo sapiens OX=9606 GN=APOA1 PE=1 SV=1
P31949	2.991681	1.580956	Protein S100-A11 OS=Homo sapiens OX=9606 GN=S100A11 PE=1 SV=2
P13647	5.044272	2.334646	Keratin, type II cytoskeletal 5 OS=Homo sapiens OX=9606 GN=KRT5 PE=1 SV=3
P01009	4.366615	2.126515	Alpha-1-antitrypsin OS=Homo sapiens OX=9606 GN=SERPINA1 PE=1 SV=3
P25325	5.365065	2.423596	3-mercaptopyruvate sulfurtransferase OS=Homo sapiens OX=9606 GN=MPST PE=1 SV=3
P09417	3.294767	1.720176	Dihydropteridine reductase OS=Homo sapiens OX=9606 GN=QDPR PE=1 SV=2
P14550	3.244313	1.697913	Aldo-keto reductase family 1 member A1 OS=Homo sapiens OX=9606 GN=AKR1A1 PE=1 SV=3
O43169	2.892794	1.532464	Cytochrome b5 type B OS=Homo sapiens OX=9606 GN=CYB5B PE=1 SV=3
P04632	2.908977	1.540512	Calpain small subunit 1 OS=Homo sapiens OX=9606 GN=CAPNS1 PE=1 SV=1
P31947	4.554423	2.187268	14-3-3 protein sigma OS=Homo sapiens OX=9606 GN=SFN PE=1 SV=1
P04080	4.189388	2.06674	Cystatin-B OS=Homo sapiens OX=9606 GN=CSTB PE=1 SV=2
P10599	3.752468	1.90784	Thioredoxin OS=Homo sapiens OX=9606 GN=TXN PE=1 SV=3
P17342	3.202348	1.67913	Atrial natriuretic peptide receptor 3 OS=Homo sapiens OX=9606 GN=NPR3 PE=1 SV=2
P16152	3.144279	1.652729	Carbonyl reductase [NADPH] 1 OS=Homo sapiens OX=9606 GN=CBR1 PE=1 SV=3
P30043	9.81281	3.294666	Flavin reductase (NADPH) OS=Homo sapiens OX=9606 GN=BLVRB PE=1 SV=3
P28907	3.144842	1.652987	ADP-ribosyl cyclase/cyclic ADP-ribose hydrolase 1 OS=Homo sapiens OX=9606 GN=CD38 PE=1 SV=2
P21926	3.916694	1.969636	CD9 antigen OS=Homo sapiens OX=9606 GN=CD9 PE=1 SV=4
P08729	5.614899	2.48926	Keratin, type II cytoskeletal 7 OS=Homo sapiens OX=9606 GN=KRT7 PE=1 SV=5
P13473	3.389507	1.761075	Lysosome-associated membrane glycoprotein 2 OS=Homo sapiens OX=9606 GN=LAMP2 PE=1 SV=2
P08697	3.270862	1.709671	Alpha-2-antiplasmin OS=Homo sapiens OX=9606 GN=SERPINF2 PE=1 SV=3
O95994	4.951961	2.308	Anterior gradient protein 2 homolog OS=Homo sapiens OX=9606 GN=AGR2 PE=1 SV=1
P35080	3.06304	1.614964	Profilin-2 OS=Homo sapiens OX=9606 GN=PFN2 PE=1 SV=3
A8MYV0	3.640166	1.864004	Doublecortin domain-containing protein 2C OS=Homo sapiens OX=9606 GN=DCDC2C PE=1 SV=4
Q9UJ83	2.851346	1.511643	2-hydroxyacyl-CoA lyase 1 OS=Homo sapiens OX=9606 GN=HACL1 PE=1 SV=2
Q9UBX7	4.425751	2.145922	Kallikrein-11 OS=Homo sapiens OX=9606 GN=KLK11 PE=1 SV=2
P42025	3.338047	1.739004	Beta-centractin OS=Homo sapiens OX=9606 GN=ACTR1B PE=1 SV=1
Q9Y394	3.622119	1.856834	Dehydrogenase/reductase SDR family member 7 OS=Homo sapiens OX=9606 GN=DHRS7 PE=1 SV=1
O95394	2.837457	1.504599	Phosphoacetylglucosamine mutase OS=Homo sapiens OX=9606 GN=PGM3 PE=1 SV=1
P07998	4.388007	2.133566	Ribonuclease pancreatic OS=Homo sapiens OX=9606 GN=RNASE1 PE=1 SV=4

P02763	6.021325	2.590081	Alpha-1-acid glycoprotein 1 OS=Homo sapiens OX=9606 GN=ORM1 PE=1 SV=2
P47895	4.387	2.133235	Aldehyde dehydrogenase family 1 member A3 OS=Homo sapiens OX=9606 GN=ALDH1A3 PE=1 SV=2
P48449	3.051359	1.609452	Lanosterol synthase OS=Homo sapiens OX=9606 GN=LSS PE=1 SV=1
O00584	3.589017	1.843589	Ribonuclease T2 OS=Homo sapiens OX=9606 GN=RNASET2 PE=1 SV=2
P06703	3.209986	1.682567	Protein S100-A6 OS=Homo sapiens OX=9606 GN=S100A6 PE=1 SV=1
P02533	9.443651	3.239345	Keratin, type I cytoskeletal 14 OS=Homo sapiens OX=9606 GN=KRT14 PE=1 SV=4
P80723	3.410645	1.770045	Brain acid soluble protein 1 OS=Homo sapiens OX=9606 GN=BASP1 PE=1 SV=2
Q06323	6.465631	2.692791	Proteasome activator complex subunit 1 OS=Homo sapiens OX=9606 GN=PSME1 PE=1 SV=1
P07099	2.842492	1.507156	Epoxide hydrolase 1 OS=Homo sapiens OX=9606 GN=EPHX1 PE=1 SV=1
P34913	7.501853	2.907247	Bifunctional epoxide hydrolase 2 OS=Homo sapiens OX=9606 GN=EPHX2 PE=1 SV=2
Q9UHY1	4.517593	2.175554	Nuclear receptor-binding protein OS=Homo sapiens OX=9606 GN=NRBP1 PE=1 SV=1
P14384	6.542975	2.709947	Carboxypeptidase M OS=Homo sapiens OX=9606 GN=CPM PE=1 SV=2
Q9GZP4	4.173018	2.061091	PITH domain-containing protein 1 OS=Homo sapiens OX=9606 GN=PITHD1 PE=1 SV=1
O43790	17.98932	4.169069	Keratin, type II cuticular Hb6 OS=Homo sapiens OX=9606 GN=KRT86 PE=1 SV=1
O43653	2.945788	1.558654	Prostate stem cell antigen OS=Homo sapiens OX=9606 GN=PSCA PE=1 SV=2
Q6UXI9	2.936953	1.55432	Nephronectin OS=Homo sapiens OX=9606 GN=NPNT PE=2 SV=3
P01834	7.940016	2.989142	Immunoglobulin kappa constant OS=Homo sapiens OX=9606 GN=IGKC PE=1 SV=2
P34096	3.757334	1.909709	Ribonuclease 4 OS=Homo sapiens OX=9606 GN=RNASE4 PE=1 SV=3
Q9HAB8	3.479017	1.79868	Phosphopantothenate--cysteine ligase OS=Homo sapiens OX=9606 GN=PPCS PE=1 SV=2
Q96DA0	2.936308	1.554003	Zymogen granule protein 16 homolog B OS=Homo sapiens OX=9606 GN=ZG16B PE=1 SV=3
Q13232	4.797361	2.262241	Nucleoside diphosphate kinase 3 OS=Homo sapiens OX=9606 GN=NME3 PE=1 SV=2
P01857	13.96745	3.803997	Immunoglobulin heavy constant gamma 1 OS=Homo sapiens OX=9606 GN=IGHG1 PE=1 SV=1
Q8NFU3	4.679489	2.226351	Thiosulfate:glutathione sulfurtransferase OS=Homo sapiens OX=9606 GN=TSTD1 PE=1 SV=3
P62942	3.578788	1.839471	Peptidyl-prolyl cis-trans isomerase FKBP1A OS=Homo sapiens OX=9606 GN=FKBP1A PE=1 SV=2
P36959	4.833271	2.273	GMP reductase 1 OS=Homo sapiens OX=9606 GN=GMPR PE=1 SV=1
P78417	3.457608	1.789774	Glutathione S-transferase omega-1 OS=Homo sapiens OX=9606 GN=GSTO1 PE=1 SV=2
O60825	3.283411	1.715195	6-phosphofructo-2-kinase/fructose-2,6-bisphosphatase 2 OS=Homo sapiens OX=9606 GN=PFKFB2 PE=1 SV=2
Q12841	12.33241	3.624383	Follistatin-related protein 1 OS=Homo sapiens OX=9606 GN=FSTL1 PE=1 SV=1
O00204	5.990286	2.582625	Sulfotransferase 2B1 OS=Homo sapiens OX=9606 GN=SULT2B1 PE=1 SV=2
Q9HAT2	2.930869	1.551329	Sialate O-acetylesterase OS=Homo sapiens OX=9606 GN=SIAE PE=1 SV=1

P30711	3.601658	1.848661	Glutathione S-transferase theta-1 OS=Homo sapiens OX=9606 GN=GSTT1 PE=1 SV=4
Q9NV96	3.351825	1.744947	Cell cycle control protein 50A OS=Homo sapiens OX=9606 GN=TMEM30A PE=1 SV=1
Q8IWA5	14.7499	3.882633	Choline transporter-like protein 2 OS=Homo sapiens OX=9606 GN=SLC44A2 PE=1 SV=3
Q13510	8.51139	3.089395	Acid ceramidase OS=Homo sapiens OX=9606 GN=ASAH1 PE=1 SV=5
O60701	4.16139	2.057065	UDP-glucose 6-dehydrogenase OS=Homo sapiens OX=9606 GN=UGDH PE=1 SV=1
Q9BTV4	2.873214	1.522665	Transmembrane protein 43 OS=Homo sapiens OX=9606 GN=TMEM43 PE=1 SV=1
Q15121	7.304161	2.868719	Astrocytic phosphoprotein PEA-15 OS=Homo sapiens OX=9606 GN=PEA15 PE=1 SV=2
P22692	2.949867	1.56065	Insulin-like growth factor-binding protein 4 OS=Homo sapiens OX=9606 GN=IGFBP4 PE=1 SV=2
P60981	6.447341	2.688704	Destrin OS=Homo sapiens OX=9606 GN=DSTN PE=1 SV=3
P0CG30	5.307007	2.407898	Glutathione S-transferase theta-2B OS=Homo sapiens OX=9606 GN=GSTT2B PE=1 SV=1
Q93099	3.465232	1.792952	Homogentisate 1,2-dioxygenase OS=Homo sapiens OX=9606 GN=HGD PE=1 SV=2
Q5T749	5.290286	2.403346	Keratinocyte proline-rich protein OS=Homo sapiens OX=9606 GN=KPRP PE=1 SV=1
P14324	3.892113	1.960554	Farnesyl pyrophosphate synthase OS=Homo sapiens OX=9606 GN=FDPS PE=1 SV=4
Q96QK1	5.425827	2.439843	Vacuolar protein sorting-associated protein 35 OS=Homo sapiens OX=9606 GN=VPS35 PE=1 SV=2
Q15323	5.754829	2.524773	Keratin, type I cuticular Ha1 OS=Homo sapiens OX=9606 GN=KRT31 PE=1 SV=3
Q9GZZ9	3.999727	1.999902	Ubiquitin-like modifier-activating enzyme 5 OS=Homo sapiens OX=9606 GN=UBA5 PE=1 SV=1
P05090	3.391137	1.761769	Apolipoprotein D OS=Homo sapiens OX=9606 GN=APOD PE=1 SV=1
P40121	4.675645	2.225165	Macrophage-capping protein OS=Homo sapiens OX=9606 GN=CAPG PE=1 SV=2
P0DOY2	5.873404	2.554197	Immunoglobulin lambda constant 2 OS=Homo sapiens OX=9606 GN=IGLC2 PE=1 SV=1
Q99988	3.438079	1.781603	Growth/differentiation factor 15 OS=Homo sapiens OX=9606 GN=GDF15 PE=1 SV=3
P19835	3.930665	1.974774	Bile salt-activated lipase OS=Homo sapiens OX=9606 GN=CEL PE=1 SV=3
P49902	5.472126	2.452101	Cytosolic purine 5'-nucleotidase OS=Homo sapiens OX=9606 GN=NT5C2 PE=1 SV=1
P09467	4.886904	2.288921	Fructose-1,6-bisphosphatase 1 OS=Homo sapiens OX=9606 GN=FBP1 PE=1 SV=5
Q6PCB0	4.814963	2.267525	von Willebrand factor A domain-containing protein 1 OS=Homo sapiens OX=9606 GN=VWA1 PE=1 SV=1
P04424	4.205495	2.072275	Argininosuccinate lyase OS=Homo sapiens OX=9606 GN=ASL PE=1 SV=4
P40189	5.994764	2.583703	Interleukin-6 receptor subunit beta OS=Homo sapiens OX=9606 GN=IL6ST PE=1 SV=2
Q9NZZ3	6.019724	2.589697	Charged multivesicular body protein 5 OS=Homo sapiens OX=9606 GN=CHMP5 PE=1 SV=1
Q53GQ0	3.084569	1.625069	Very-long-chain 3-oxoacyl-CoA reductase OS=Homo sapiens OX=9606 GN=HSD17B12 PE=1 SV=2

P98095	8.511594	3.089429	Fibulin-2 OS=Homo sapiens OX=9606 GN=FBLN2 PE=1 SV=2
Q53GD3	5.006118	2.323692	Choline transporter-like protein 4 OS=Homo sapiens OX=9606 GN=SLC44A4 PE=1 SV=2
P49588	6.566496	2.715124	Alanine--tRNA ligase, cytoplasmic OS=Homo sapiens OX=9606 GN=AARS1 PE=1 SV=2
P01859	5.82333	2.541844	Immunoglobulin heavy constant gamma 2 OS=Homo sapiens OX=9606 GN=IGHG2 PE=1 SV=2
Q9Y2S2	4.769614	2.253872	Lambda-crystallin homolog OS=Homo sapiens OX=9606 GN=CRYL1 PE=1 SV=3
O15296	10.91129	3.44775	Polyunsaturated fatty acid lipoxygenase ALOX15B OS=Homo sapiens OX=9606 GN=ALOX15B PE=1 SV=3
P21953	3.767789	1.913718	2-oxoisovalerate dehydrogenase subunit beta, mitochondrial OS=Homo sapiens OX=9606 GN=BCKDHB PE=1 SV=2
O43278	9.061276	3.179714	Kunitz-type protease inhibitor 1 OS=Homo sapiens OX=9606 GN=SPINT1 PE=1 SV=2
Q7L1Q6	4.193096	2.068016	eIF5-mimic protein 2 OS=Homo sapiens OX=9606 GN=BZW1 PE=1 SV=1
P19652	10.97657	3.456355	Alpha-1-acid glycoprotein 2 OS=Homo sapiens OX=9606 GN=ORM2 PE=1 SV=2
Q6YN16	23.74826	4.56975	Hydroxysteroid dehydrogenase-like protein 2 OS=Homo sapiens OX=9606 GN=HSDL2 PE=1 SV=1
Q9UBG3	6.931709	2.793211	Cornulin OS=Homo sapiens OX=9606 GN=CRNN PE=1 SV=1
Q8WVQ1	3.071258	1.61883	Soluble calcium-activated nucleotidase 1 OS=Homo sapiens OX=9606 GN=CANT1 PE=1 SV=1
P40763	3.675835	1.878072	Signal transducer and activator of transcription 3 OS=Homo sapiens OX=9606 GN=STAT3 PE=1 SV=2
Q6UX06	16.67178	4.059336	Olfactomedin-4 OS=Homo sapiens OX=9606 GN=OLFM4 PE=1 SV=1
Q13557	5.032037	2.331143	Calcium/calmodulin-dependent protein kinase type II subunit delta OS=Homo sapiens OX=9606 GN=CAMK2D PE=1 SV=3
Q15436	7.235179	2.855029	Protein transport protein Sec23A OS=Homo sapiens OX=9606 GN=SEC23A PE=1 SV=2
Q14764	4.452594	2.154646	Major vault protein OS=Homo sapiens OX=9606 GN=MVP PE=1 SV=4
O43615	7.069268	2.821561	Mitochondrial import inner membrane translocase subunit TIM44 OS=Homo sapiens OX=9606 GN=TIMM44 PE=1 SV=2
O14638	5.377384	2.426905	Ectonucleotide pyrophosphatase/phosphodiesterase family member 3 OS=Homo sapiens OX=9606 GN=ENPP3 PE=1 SV=2
O00194	6.411869	2.680745	Ras-related protein Rab-27B OS=Homo sapiens OX=9606 GN=RAB27B PE=1 SV=4
Q93052	4.685122	2.228087	Lipoma-preferred partner OS=Homo sapiens OX=9606 GN=LPP PE=1 SV=1
Q09666	8.727637	3.125591	Neuroblast differentiation-associated protein AHNAK OS=Homo sapiens OX=9606 GN=AHNAK PE=1 SV=2
P30520	3.398442	1.764873	Adenylosuccinate synthetase isozyme 2 OS=Homo sapiens OX=9606 GN=ADSS2 PE=1 SV=3
P14735	4.664742	2.221797	Insulin-degrading enzyme OS=Homo sapiens OX=9606 GN=IDE PE=1 SV=4
O95716	13.76307	3.782731	Ras-related protein Rab-3D OS=Homo sapiens OX=9606 GN=RAB3D PE=1 SV=1
Q8IYS1	9.891565	3.306199	Xaa-Arg dipeptidase OS=Homo sapiens OX=9606 GN=PM20D2 PE=1 SV=2
O00560	3.993259	1.997566	Syntenin-1 OS=Homo sapiens OX=9606 GN=SDCBP PE=1 SV=1
Q687X5	4.808325	2.265534	Metalloreductase STEAP4 OS=Homo sapiens OX=9606 GN=STEAP4 PE=1 SV=1
P51812	3.471709	1.795646	Ribosomal protein S6 kinase alpha-3 OS=Homo sapiens OX=9606 GN=RPS6KA3 PE=1 SV=1

O75351	3.04301	1.605499	Vacuolar protein sorting-associated protein 4B OS=Homo sapiens OX=9606 GN=VPS4B PE=1 SV=2
P53004	7.653299	2.936082	Biliverdin reductase A OS=Homo sapiens OX=9606 GN=BLVRA PE=1 SV=2
Q9Y625	3.596652	1.846654	Glypican-6 OS=Homo sapiens OX=9606 GN=GPC6 PE=1 SV=1
P12268	3.395634	1.763681	Inosine-5'-monophosphate dehydrogenase 2 OS=Homo sapiens OX=9606 GN=IMPDH2 PE=1 SV=2
Q96DG6	5.304019	2.407086	Carboxymethylenebutenolidase homolog OS=Homo sapiens OX=9606 GN=CMBL PE=1 SV=1
Q08209	15.13735	3.920041	Serine/threonine-protein phosphatase 2B catalytic subunit alpha isoform OS=Homo sapiens OX=9606 GN=PPP3CA PE=1 SV=1
P30740	13.76238	3.782658	Leukocyte elastase inhibitor OS=Homo sapiens OX=9606 GN=SERPINB1 PE=1 SV=1
P54108	20.90321	4.385652	Cysteine-rich secretory protein 3 OS=Homo sapiens OX=9606 GN=CRISP3 PE=1 SV=1
Q14894	4.308995	2.107351	Ketimine reductase mu-crystallin OS=Homo sapiens OX=9606 GN=CRYM PE=1 SV=1
O00462	3.377337	1.755886	Beta-mannosidase OS=Homo sapiens OX=9606 GN=MANBA PE=1 SV=3
Q13449	11.94574	3.578424	Limbic system-associated membrane protein OS=Homo sapiens OX=9606 GN=LSAMP PE=1 SV=2
P00167	4.900242	2.292853	Cytochrome b5 OS=Homo sapiens OX=9606 GN=CYB5A PE=1 SV=2
P01876	10.73449	3.424181	Immunoglobulin heavy constant alpha 1 OS=Homo sapiens OX=9606 GN=IGHA1 PE=1 SV=2
P16035	3.482143	1.799975	Metalloproteinase inhibitor 2 OS=Homo sapiens OX=9606 GN=TIMP2 PE=1 SV=2
O15355	4.47717	2.162587	Protein phosphatase 1G OS=Homo sapiens OX=9606 GN=PPM1G PE=1 SV=1
P24593	4.219518	2.077078	Insulin-like growth factor-binding protein 5 OS=Homo sapiens OX=9606 GN=IGFBP5 PE=1 SV=1
Q9UBC9	92.16379	6.526128	Small proline-rich protein 3 OS=Homo sapiens OX=9606 GN=SPRR3 PE=1 SV=2
Q86YZ3	26.97876	4.753752	Hornerin OS=Homo sapiens OX=9606 GN=HRNR PE=1 SV=2

91 Down-regulated

ID	*FC*	*Log2FC*	*Description*
P49913	0.184725	-2.43655	Cathelicidin antimicrobial peptide OS=Homo sapiens OX=9606 GN=CAMP PE=1 SV=1
P56851	0.314584	-1.66848	Epididymal secretory protein E3-beta OS=Homo sapiens OX=9606 GN=EDDM3B PE=1 SV=2
Q96RL7	0.351559	-1.50816	Vacuolar protein sorting-associated protein 13A OS=Homo sapiens OX=9606 GN=VPS13A PE=1 SV=2
Q9Y2B4	0.233706	-2.09723	TP53-target gene 5 protein OS=Homo sapiens OX=9606 GN=TP53TG5 PE=1 SV=1
P29401	0.180725	-2.46814	Transketolase OS=Homo sapiens OX=9606 GN=TKT PE=1 SV=3
P53602	0.083905	-3.57511	Diphosphomevalonate decarboxylase OS=Homo sapiens OX=9606 GN=MVD PE=1 SV=1
Q08830	0.296403	-1.75437	Fibrinogen-like protein 1 OS=Homo sapiens OX=9606 GN=FGL1 PE=1 SV=3

Q9NZ01	0.268677	-1.89606	Very-long-chain enoyl-CoA reductase OS=Homo sapiens OX=9606 GN=TECR PE=1 SV=1
Q9Y371	0.338111	-1.56443	Endophilin-B1 OS=Homo sapiens OX=9606 GN=SH3GLB1 PE=1 SV=1
P06748	0.092463	-3.43498	Nucleophosmin OS=Homo sapiens OX=9606 GN=NPM1 PE=1 SV=2
Q92743	0.21481	-2.21887	Serine protease HTRA1 OS=Homo sapiens OX=9606 GN=HTRA1 PE=1 SV=1
Q9P260	0.13543	-2.88438	RAB11-binding protein RELCH OS=Homo sapiens OX=9606 GN=RELCH PE=1 SV=2
Q6UW15	0.320571	-1.64128	Regenerating islet-derived protein 3-gamma OS=Homo sapiens OX=9606 GN=REG3G PE=1 SV=1
Q96P26	0.179233	-2.48009	Cytosolic 5'-nucleotidase 1B OS=Homo sapiens OX=9606 GN=NT5C1B PE=2 SV=2
P22626	0.12825	-2.96297	Heterogeneous nuclear ribonucleoproteins A2/B1 OS=Homo sapiens OX=9606 GN=HNRNPA2B1 PE=1 SV=2
O75556	0.125315	-2.99637	Mammaglobin-B OS=Homo sapiens OX=9606 GN=SCGB2A1 PE=1 SV=1
O95969	0.029697	-5.07355	Secretoglobin family 1D member 2 OS=Homo sapiens OX=9606 GN=SCGB1D2 PE=2 SV=1
P07910	0.34103	-1.55203	Heterogeneous nuclear ribonucleoproteins C1/C2 OS=Homo sapiens OX=9606 GN=HNRNPC PE=1 SV=4
Q9UK41	0.07587	-3.72033	Vacuolar protein sorting-associated protein 28 homolog OS=Homo sapiens OX=9606 GN=VPS28 PE=1 SV=1
O94973	0.227329	-2.13714	AP-2 complex subunit alpha-2 OS=Homo sapiens OX=9606 GN=AP2A2 PE=1 SV=2
P21810	0.276078	-1.85685	Biglycan OS=Homo sapiens OX=9606 GN=BGN PE=1 SV=2
Q86Y82	0.259218	-1.94776	Syntaxin-12 OS=Homo sapiens OX=9606 GN=STX12 PE=1 SV=1
O75787	0.347087	-1.52663	Renin receptor OS=Homo sapiens OX=9606 GN=ATP6AP2 PE=1 SV=2
O14967	0.299815	-1.73786	Calmegin OS=Homo sapiens OX=9606 GN=CLGN PE=1 SV=1
P06737	0.234037	-2.09519	Glycogen phosphorylase, liver form OS=Homo sapiens OX=9606 GN=PYGL PE=1 SV=4
P07738	0.25223	-1.98719	Bisphosphoglycerate mutase OS=Homo sapiens OX=9606 GN=BPGM PE=1 SV=2
P26583	0.321142	-1.63872	High mobility group protein B2 OS=Homo sapiens OX=9606 GN=HMGB2 PE=1 SV=2
Q8NF91	0.246119	-2.02257	Nesprin-1 OS=Homo sapiens OX=9606 GN=SYNE1 PE=1 SV=4
P51854	0.181782	-2.45972	Transketolase-like protein 1 OS=Homo sapiens OX=9606 GN=TKTL1 PE=1 SV=2
Q9UKA9	0.062469	-4.0007	Polypyrimidine tract-binding protein 2 OS=Homo sapiens OX=9606 GN=PTBP2 PE=1 SV=1
Q5XKP0	0.335131	-1.5772	MICOS complex subunit MIC13 OS=Homo sapiens OX=9606 GN=MICOS13 PE=1 SV=1
Q9NP58	0.102978	-3.27959	ATP-binding cassette sub-family B member 6 OS=Homo sapiens OX=9606 GN=ABCB6 PE=1 SV=1
Q9Y2T7	0.10179	-3.29633	Y-box-binding protein 2 OS=Homo sapiens OX=9606 GN=YBX2 PE=1 SV=2
Q14103	0.264284	-1.91984	Heterogeneous nuclear ribonucleoprotein D0 OS=Homo sapiens OX=9606 GN=HNRNPD PE=1 SV=1
P15170	0.337941	-1.56516	Eukaryotic peptide chain release factor GTP-binding subunit ERF3A OS=Homo sapiens OX=9606 GN=GSPT1 PE=1 SV=1
P0C8F1	0.179638	-2.47684	Prostate and testis expressed protein 4 OS=Homo sapiens OX=9606 GN=PATE4 PE=2 SV=2
P35813	0.343035	-1.54357	Protein phosphatase 1A OS=Homo sapiens OX=9606 GN=PPM1A PE=1 SV=1

O14562	0.180931	-2.46649	Ubiquitin domain-containing protein UBFD1 OS=Homo sapiens OX=9606 GN=UBFD1 PE=1 SV=2
P45877	0.316995	-1.65747	Peptidyl-prolyl cis-trans isomerase C OS=Homo sapiens OX=9606 GN=PPIC PE=1 SV=1
Q7Z304	0.121704	-3.03855	MAM domain-containing protein 2 OS=Homo sapiens OX=9606 GN=MAMDC2 PE=1 SV=3
O43854	0.17673	-2.50038	EGF-like repeat and discoidin I-like domain-containing protein 3 OS=Homo sapiens OX=9606 GN=EDIL3 PE=1 SV=1
P63279	0.323751	-1.62704	SUMO-conjugating enzyme UBC9 OS=Homo sapiens OX=9606 GN=UBE2I PE=1 SV=1
Q9Y333	0.340048	-1.55619	U6 snRNA-associated Sm-like protein LSm2 OS=Homo sapiens OX=9606 GN=LSM2 PE=1 SV=1
O75663	0.3292	-1.60296	TIP41-like protein OS=Homo sapiens OX=9606 GN=TIPRL PE=1 SV=2
Q86X55	0.33805	-1.56469	Histone-arginine methyltransferase CARM1 OS=Homo sapiens OX=9606 GN=CARM1 PE=1 SV=3
Q9NRY7	0.141667	-2.81942	Phospholipid scramblase 2 OS=Homo sapiens OX=9606 GN=PLSCR2 PE=1 SV=2
Q8NFH4	0.302586	-1.72458	Nucleoporin Nup37 OS=Homo sapiens OX=9606 GN=NUP37 PE=1 SV=1
Q68CQ1	0.287871	-1.79651	Maestro heat-like repeat-containing protein family member 7 OS=Homo sapiens OX=9606 GN=MROH7 PE=2 SV=4
O14787-2	0.208128	-2.26446	Isoform 2 of Transportin-2 OS=Homo sapiens OX=9606 GN=TNPO2
Q8TCS8	0.199086	-2.32854	Polyribonucleotide nucleotidyltransferase 1, mitochondrial OS=Homo sapiens OX=9606 GN=PNPT1 PE=1 SV=2
Q6PI48	0.32126	-1.63819	Aspartate--tRNA ligase, mitochondrial OS=Homo sapiens OX=9606 GN=DARS2 PE=1 SV=1
P62318	0.178543	-2.48566	Small nuclear ribonucleoprotein Sm D3 OS=Homo sapiens OX=9606 GN=SNRPD3 PE=1 SV=1
Q99523	0.314698	-1.66796	Sortilin OS=Homo sapiens OX=9606 GN=SORT1 PE=1 SV=3
P52272	0.165726	-2.59313	Heterogeneous nuclear ribonucleoprotein M OS=Homo sapiens OX=9606 GN=HNRNPM PE=1 SV=3
Q7RTN6	0.291674	-1.77757	STE20-related kinase adapter protein alpha OS=Homo sapiens OX=9606 GN=STRADA PE=1 SV=1
Q8NFW8	0.213526	-2.22752	N-acylneuraminate cytidylyltransferase OS=Homo sapiens OX=9606 GN=CMAS PE=1 SV=2
P14543	0.186207	-2.42502	Nidogen-1 OS=Homo sapiens OX=9606 GN=NID1 PE=1 SV=3
Q8IVD9	0.299748	-1.73818	NudC domain-containing protein 3 OS=Homo sapiens OX=9606 GN=NUDCD3 PE=1 SV=3
Q9BW27	0.218312	-2.19553	Nuclear pore complex protein Nup85 OS=Homo sapiens OX=9606 GN=NUP85 PE=1 SV=1
Q8N4T8	0.350555	-1.51229	3-oxoacyl-[acyl-carrier-protein] reductase OS=Homo sapiens OX=9606 GN=CBR4 PE=1 SV=3
Q92544	0.139078	-2.84604	Transmembrane 9 superfamily member 4 OS=Homo sapiens OX=9606 GN=TM9SF4 PE=1 SV=2
Q9GZY8	0.26478	-1.91714	Mitochondrial fission factor OS=Homo sapiens OX=9606 GN=MFF PE=1 SV=1
Q9Y5U8	0.343091	-1.54334	Mitochondrial pyruvate carrier 1 OS=Homo sapiens OX=9606 GN=MPC1 PE=1 SV=1
Q9UHQ4	0.151226	-2.72522	B-cell receptor-associated protein 29 OS=Homo sapiens OX=9606 GN=BCAP29 PE=1 SV=2
P49788	0.341521	-1.54995	Retinoic acid receptor responder protein 1 OS=Homo sapiens OX=9606 GN=RARRES1 PE=1 SV=2

Q9Y6D5	0.187929	-2.41174	Brefeldin A-inhibited guanine nucleotide-exchange protein 2 OS=Homo sapiens OX=9606 GN=ARFGEF2 PE=1 SV=3
O95139	0.305045	-1.7129	NADH dehydrogenase [ubiquinone] 1 beta subcomplex subunit 6 OS=Homo sapiens OX=9606 GN=NDUFB6 PE=1 SV=3
Q9UMQ6	0.226308	-2.14364	Calpain-11 OS=Homo sapiens OX=9606 GN=CAPN11 PE=2 SV=2
Q9NWU2	0.299102	-1.74129	Glucose-induced degradation protein 8 homolog OS=Homo sapiens OX=9606 GN=GID8 PE=1 SV=1
P62314	0.35024	-1.51358	Small nuclear ribonucleoprotein Sm D1 OS=Homo sapiens OX=9606 GN=SNRPD1 PE=1 SV=1
Q8TEX9	0.150366	-2.73344	Importin-4 OS=Homo sapiens OX=9606 GN=IPO4 PE=1 SV=2
Q8TAP6	0.301591	-1.72933	Centrosomal protein of 76 kDa OS=Homo sapiens OX=9606 GN=CEP76 PE=1 SV=1
P51991	0.193982	-2.36601	Heterogeneous nuclear ribonucleoprotein A3 OS=Homo sapiens OX=9606 GN=HNRNPA3 PE=1 SV=2
Q9P0J7	0.127212	-2.97469	E3 ubiquitin-protein ligase KCMF1 OS=Homo sapiens OX=9606 GN=KCMF1 PE=1 SV=2
Q15813	0.346671	-1.52836	Tubulin-specific chaperone E OS=Homo sapiens OX=9606 GN=TBCE PE=1 SV=1
Q5RHP9	0.195869	-2.35204	Glutamate-rich protein 3 OS=Homo sapiens OX=9606 GN=ERICH3 PE=1 SV=1
Q9NQC8	0.318426	-1.65097	Intraflagellar transport protein 46 homolog OS=Homo sapiens OX=9606 GN=IFT46 PE=1 SV=1
Q7Z3C6	0.191404	-2.38531	Autophagy-related protein 9A OS=Homo sapiens OX=9606 GN=ATG9A PE=1 SV=3
Q9NQ36	0.241418	-2.05039	Signal peptide, CUB and EGF-like domain-containing protein 2 OS=Homo sapiens OX=9606 GN=SCUBE2 PE=1 SV=3
Q9BV10	0.232494	-2.10473	Dol-P-Man:Man(7)GlcNAc(2)-PP-Dol alpha-1,6-mannosyltransferase OS=Homo sapiens OX=9606 GN=ALG12 PE=1 SV=1
Q03169	0.12549	-2.99436	Tumor necrosis factor alpha-induced protein 2 OS=Homo sapiens OX=9606 GN=TNFAIP2 PE=1 SV=2
Q8NE86	0.30885	-1.69502	Calcium uniporter protein, mitochondrial OS=Homo sapiens OX=9606 GN=MCU PE=1 SV=1
P62263	0.316959	-1.65763	40S ribosomal protein S14 OS=Homo sapiens OX=9606 GN=RPS14 PE=1 SV=3
Q70HW3	0.206635	-2.27484	S-adenosylmethionine mitochondrial carrier protein OS=Homo sapiens OX=9606 GN=SLC25A26 PE=1 SV=2
Q8WXF7	0.351176	-1.50973	Atlastin-1 OS=Homo sapiens OX=9606 GN=ATL1 PE=1 SV=1
Q92734	0.341121	-1.55164	Protein TFG OS=Homo sapiens OX=9606 GN=TFG PE=1 SV=2
Q8NEL9	0.318269	-1.65168	Phospholipase DDHD1 OS=Homo sapiens OX=9606 GN=DDHD1 PE=1 SV=2
Q9NQH7	0.293421	-1.76896	Xaa-Pro aminopeptidase 3 OS=Homo sapiens OX=9606 GN=XPNPEP3 PE=1 SV=1
O14828	0.274974	-1.86263	Secretory carrier-associated membrane protein 3 OS=Homo sapiens OX=9606 GN=SCAMP3 PE=1 SV=3
P50454	0.259122	-1.94829	Serpin H1 OS=Homo sapiens OX=9606 GN=SERPINH1 PE=1 SV=2
O75955	0.155009	-2.68958	Flotillin-1 OS=Homo sapiens OX=9606 GN=FLOT1 PE=1 SV=3
