# Supplementary material for: Molecular Remodeling of the Sperm Proteome Following Varicocele Sclero-Embolization: Implications for Semen Quality Improvement
Source: Proteomes. 2025 Jul 15;13(3):34. doi: 10.3390/proteomes13030034 (PMC12286009; doi:10.3390/proteomes13030034)
Supplement: Supplementary file 1 [file proteomes-13-00034-s001.zip › Suppl file S4 Diff.expressed POST6M_PRE.pdf]

DIFFERENTIAL PROTEIN EXPRESSION BASED ON FOLD CHANGE POST 6M / PRE

Increase of expression = Log2(Fold Change) > 1.5

Decrease of expression = Log2(Fold Change) < -1.5

174 Up-regulated

ID	FC	Log2FC	Description
Q02383	4.85506765	2.279491398	Semenogelin-2 OS=Homo sapiens OX=9606 GN=SEMG2 PE=1 SV=1
Q8WZ59	3.667237973	1.874693888	Transmembrane protein 190 OS=Homo sapiens OX=9606 GN=TMEM190 PE=1 SV=1
P15309	5.707421991	2.512839236	Prostatic acid phosphatase OS=Homo sapiens OX=9606 GN=ACP3 PE=1 SV=3
P02788	3.530358924	1.819814867	Lactotransferrin OS=Homo sapiens OX=9606 GN=LTF PE=1 SV=6
P08118	9.017697519	3.172759118	Beta-microseminoprotein OS=Homo sapiens OX=9606 GN=MSMB PE=1 SV=1
P07288	9.926220316	3.311244476	Prostate-specific antigen OS=Homo sapiens OX=9606 GN=KLK3 PE=1 SV=2
P17174	3.277294479	1.712505311	Aspartate aminotransferase, cytoplasmic OS=Homo sapiens OX=9606 GN=GOT1 PE=1 SV=3
P15924	3.825560894	1.935671288	Desmoplakin OS=Homo sapiens OX=9606 GN=DSP PE=1 SV=3
P61916	3.118029994	1.640634806	NPC intracellular cholesterol transporter 2 OS=Homo sapiens OX=9606 GN=NPC2 PE=1 SV=1
P25311	8.966417532	3.164531682	Zinc-alpha-2-glycoprotein OS=Homo sapiens OX=9606 GN=AZGP1 PE=1 SV=2
Q9UIA9	6.415806214	2.681630567	Exportin-7 OS=Homo sapiens OX=9606 GN=XPO7 PE=1 SV=3
P61769	3.527114035	1.81848822	Beta-2-microglobulin OS=Homo sapiens OX=9606 GN=B2M PE=1 SV=1
P09466	4.357356706	2.123453221	Glycodelin OS=Homo sapiens OX=9606 GN=PAEP PE=1 SV=2
Q6W4X9	7.362102696	2.880117874	Mucin-6 OS=Homo sapiens OX=9606 GN=MUC6 PE=1 SV=3
Q14508	5.977175987	2.579464021	WAP four-disulfide core domain protein 2 OS=Homo sapiens OX=9606 GN=WFDC2 PE=1 SV=2
P01034	5.540488583	2.470013205	Cystatin-C OS=Homo sapiens OX=9606 GN=CST3 PE=1 SV=1
P05154	6.109137093	2.610968616	Plasma serine protease inhibitor OS=Homo sapiens OX=9606 GN=SERPINA5 PE=1 SV=3
Q8TEQ8	3.740323653	1.903163113	GPI ethanolamine phosphate transferase 3 OS=Homo sapiens OX=9606 GN=PIGO PE=1 SV=3
P20151	3.357332423	1.747315391	Kallikrein-2 OS=Homo sapiens OX=9606 GN=KLK2 PE=1 SV=1
Q8TF09	2.870126139	1.521114143	Dynein light chain roadblock-type 2 OS=Homo sapiens OX=9606 GN=DYNLRB2 PE=1 SV=1
Q8N6Q3	3.688013001	1.882843742	CD177 antigen OS=Homo sapiens OX=9606 GN=CD177 PE=1 SV=2
P30153	5.200085138	2.378535244	Serine/threonine-protein phosphatase 2A 65 kDa regulatory subunit A alpha isoform OS=Homo sapiens OX=9606 GN=PPP2R1A PE=1 SV=4
Q9NWV4	19.56255907	4.290023203	CXXC motif containing zinc binding protein OS=Homo sapiens OX=9606 GN=CZIB PE=1 SV=1
Q8N807	5.690944545	2.508668121	Protein disulfide-isomerase-like protein of the testis OS=Homo sapiens OX=9606 GN=PDILT PE=1 SV=2

P13646	6.489716547	2.698155467	Keratin, type I cytoskeletal 13 OS=Homo sapiens OX=9606 GN=KRT13 PE=1 SV=4
P15144	3.875071482	1.954222924	Aminopeptidase N OS=Homo sapiens OX=9606 GN=ANPEP PE=1 SV=4
P68871	3.454590282	1.788514616	Hemoglobin subunit beta OS=Homo sapiens OX=9606 GN=HBB PE=1 SV=2
Q8NDM7	3.261593003	1.705576767	Cilia- and flagella-associated protein 43 OS=Homo sapiens OX=9606 GN=CFAP43 PE=1 SV=3
O94886	3.049705507	1.608669937	CSC1-like protein 1 OS=Homo sapiens OX=9606 GN=TMEM63A PE=1 SV=3
P01036	5.642540209	2.496344794	Cystatin-S OS=Homo sapiens OX=9606 GN=CST4 PE=1 SV=3
P11766	3.277170101	1.712450558	Alcohol dehydrogenase class-3 OS=Homo sapiens OX=9606 GN=ADH5 PE=1 SV=4
Q04760	4.169574289	2.059900093	Lactoylglutathione lyase OS=Homo sapiens OX=9606 GN=GLO1 PE=1 SV=4
Q99985	2.989681165	1.579991636	Semaphorin-3C OS=Homo sapiens OX=9606 GN=SEMA3C PE=2 SV=2
P02751	3.270655878	1.709579974	Fibronectin OS=Homo sapiens OX=9606 GN=FN1 PE=1 SV=5
P12429	3.822225041	1.934412722	Annexin A3 OS=Homo sapiens OX=9606 GN=ANXA3 PE=1 SV=3
P31949	3.560752318	1.832182087	Protein S100-A11 OS=Homo sapiens OX=9606 GN=S100A11 PE=1 SV=2
P01009	6.427038831	2.684154189	Alpha-1-antitrypsin OS=Homo sapiens OX=9606 GN=SERPINA1 PE=1 SV=3
P02790	3.372430318	1.753788634	Hemopexin OS=Homo sapiens OX=9606 GN=HPX PE=1 SV=2
P14550	2.983867114	1.577183287	Aldo-keto reductase family 1 member A1 OS=Homo sapiens OX=9606 GN=AKR1A1 PE=1 SV=3
P31947	3.916210317	1.969458246	14-3-3 protein sigma OS=Homo sapiens OX=9606 GN=SFN PE=1 SV=1
P04080	4.330428881	2.114509915	Cystatin-B OS=Homo sapiens OX=9606 GN=CSTB PE=1 SV=2
P07355	37.93105672	5.245307659	Annexin A2 OS=Homo sapiens OX=9606 GN=ANXA2 PE=1 SV=2
P55157	3.491164841	1.803708478	Microsomal triglyceride transfer protein large subunit OS=Homo sapiens OX=9606 GN=MTTP PE=1 SV=1
P10599	3.725886013	1.897583538	Thioredoxin OS=Homo sapiens OX=9606 GN=TXN PE=1 SV=3
A6NCJ1	3.266469902	1.707732347	Uncharacterized protein C19orf71 OS=Homo sapiens OX=9606 GN=C19orf71 PE=4 SV=2
P07858	3.017493375	1.593350604	Cathepsin B OS=Homo sapiens OX=9606 GN=CTSB PE=1 SV=3
O75439	3.045795419	1.606819042	Mitochondrial-processing peptidase subunit beta OS=Homo sapiens OX=9606 GN=PMPCB PE=1 SV=2
P30043	6.609193341	2.7244742	Flavin reductase (NADPH) OS=Homo sapiens OX=9606 GN=BLVRB PE=1 SV=3
P28907	3.636754857	1.862651681	ADP-ribosyl cyclase/cyclic ADP-ribose hydrolase 1 OS=Homo sapiens OX=9606 GN=CD38 PE=1 SV=2
P21926	12.80683553	3.678842136	CD9 antigen OS=Homo sapiens OX=9606 GN=CD9 PE=1 SV=4
P13473	4.69379713	2.230755489	Lysosome-associated membrane glycoprotein 2 OS=Homo sapiens OX=9606 GN=LAMP2 PE=1 SV=2
O75380	2.893468327	1.532799852	NADH dehydrogenase [ubiquinone] iron-sulfur protein 6, mitochondrial OS=Homo sapiens OX=9606 GN=NDUFS6 PE=1 SV=1
P55884	2.979174014	1.574912394	Eukaryotic translation initiation factor 3 subunit B OS=Homo sapiens OX=9606 GN=EIF3B PE=1 SV=3
P08697	6.595705489	2.721526981	Alpha-2-antiplasmin OS=Homo sapiens OX=9606 GN=SERPINF2 PE=1 SV=3

O95994	3.241181872	1.696519977	Anterior gradient protein 2 homolog OS=Homo sapiens OX=9606 GN=AGR2 PE=1 SV=1
P35080	2.882372446	1.527256766	Profilin-2 OS=Homo sapiens OX=9606 GN=PFN2 PE=1 SV=3
Q9UBX7	5.793547582	2.534447029	Kallikrein-11 OS=Homo sapiens OX=9606 GN=KLK11 PE=1 SV=2
Q14393	2.975818759	1.573286662	Growth arrest-specific protein 6 OS=Homo sapiens OX=9606 GN=GAS6 PE=1 SV=3
P07998	8.762685172	3.131373026	Ribonuclease pancreatic OS=Homo sapiens OX=9606 GN=RNASE1 PE=1 SV=4
Q9Y5Y5	11.2817846	3.495923392	Peroxisomal membrane protein PEX16 OS=Homo sapiens OX=9606 GN=PEX16 PE=1 SV=2
P02763	52.09789194	5.703153092	Alpha-1-acid glycoprotein 1 OS=Homo sapiens OX=9606 GN=ORM1 PE=1 SV=2
P47895	4.093848351	2.033457661	Aldehyde dehydrogenase family 1 member A3 OS=Homo sapiens OX=9606 GN=ALDH1A3 PE=1 SV=2
P48449	3.842777776	1.942149549	Lanosterol synthase OS=Homo sapiens OX=9606 GN=LSS PE=1 SV=1
O00584	7.471932217	2.901481367	Ribonuclease T2 OS=Homo sapiens OX=9606 GN=RNASET2 PE=1 SV=2
Q12904	2.884473357	1.528307938	Aminoacyl tRNA synthase complex-interacting multifunctional protein 1 OS=Homo sapiens OX=9606 GN=AIMP1 PE=1 SV=2
P06703	3.467211726	1.793775938	Protein S100-A6 OS=Homo sapiens OX=9606 GN=S100A6 PE=1 SV=1
P80723	4.390812863	2.134488048	Brain acid soluble protein 1 OS=Homo sapiens OX=9606 GN=BASP1 PE=1 SV=2
Q06323	4.561144458	2.189395863	Proteasome activator complex subunit 1 OS=Homo sapiens OX=9606 GN=PSME1 PE=1 SV=1
P34913	4.640731493	2.214352228	Bifunctional epoxide hydrolase 2 OS=Homo sapiens OX=9606 GN=EPHX2 PE=1 SV=2
Q9UHY1	7.40476162	2.888453292	Nuclear receptor-binding protein OS=Homo sapiens OX=9606 GN=NRBP1 PE=1 SV=1
P14384	5.242185235	2.390168333	Carboxypeptidase M OS=Homo sapiens OX=9606 GN=CPM PE=1 SV=2
Q9GZP4	4.340817826	2.117966877	PITH domain-containing protein 1 OS=Homo sapiens OX=9606 GN=PITHD1 PE=1 SV=1
O43653	8.096042329	3.017216833	Prostate stem cell antigen OS=Homo sapiens OX=9606 GN=PSCA PE=1 SV=2
Q9UPN3	3.074080898	1.620155132	Microtubule-actin cross-linking factor 1, isoforms 1/2/3/5 OS=Homo sapiens OX=9606 GN=MACF1 PE=1 SV=4
Q6UXI9	3.124105637	1.643443237	Nephronectin OS=Homo sapiens OX=9606 GN=NPNT PE=2 SV=3
Q99519	4.216476648	2.076037965	Sialidase-1 OS=Homo sapiens OX=9606 GN=NEU1 PE=1 SV=1
P01834	16.40794734	4.036322862	Immunoglobulin kappa constant OS=Homo sapiens OX=9606 GN=IGKC PE=1 SV=2
P34096	4.784619901	2.258404319	Ribonuclease 4 OS=Homo sapiens OX=9606 GN=RNASE4 PE=1 SV=3
Q13438	3.749305645	1.90662344	Protein OS-9 OS=Homo sapiens OX=9606 GN=OS9 PE=1 SV=1
P48444	3.107676673	1.635836411	Coatomer subunit delta OS=Homo sapiens OX=9606 GN=ARCN1 PE=1 SV=1
Q9HAB8	3.06033738	1.613690708	Phosphopantothenate--cysteine ligase OS=Homo sapiens OX=9606 GN=PPCS PE=1 SV=2
Q06481	3.519057187	1.815188959	Amyloid beta precursor like protein 2 OS=Homo sapiens OX=9606 GN=APLP2 PE=1 SV=2

P18065	8.658722797	3.114154236	Insulin-like growth factor-binding protein 2 OS=Homo sapiens OX=9606 GN=IGFBP2 PE=1 SV=2
Q96DA0	3.428547892	1.777597675	Zymogen granule protein 16 homolog B OS=Homo sapiens OX=9606 GN=ZG16B PE=1 SV=3
O75503	4.223820566	2.078548548	Ceroid-lipofuscinosis neuronal protein 5 OS=Homo sapiens OX=9606 GN=CLN5 PE=1 SV=2
P08294	4.755825016	2.249695634	Extracellular superoxide dismutase [Cu-Zn] OS=Homo sapiens OX=9606 GN=SOD3 PE=1 SV=2
Q13232	5.330589134	2.414294988	Nucleoside diphosphate kinase 3 OS=Homo sapiens OX=9606 GN=NME3 PE=1 SV=2
P01857	18.47959016	4.207860856	Immunoglobulin heavy constant gamma 1 OS=Homo sapiens OX=9606 GN=IGHG1 PE=1 SV=1
Q9Y3A5	4.139933679	2.049607656	Ribosome maturation protein SBDS OS=Homo sapiens OX=9606 GN=SBDS PE=1 SV=4
P11217	3.196150377	1.676335288	Glycogen phosphorylase, muscle form OS=Homo sapiens OX=9606 GN=PYGM PE=1 SV=6
P62942	3.195699843	1.676131909	Peptidyl-prolyl cis-trans isomerase FKBP1A OS=Homo sapiens OX=9606 GN=FKBP1A PE=1 SV=2
P36959	3.808250457	1.929128363	GMP reductase 1 OS=Homo sapiens OX=9606 GN=GMPR PE=1 SV=1
P61960	3.544700393	1.825663692	Ubiquitin-fold modifier 1 OS=Homo sapiens OX=9606 GN=UFM1 PE=1 SV=1
Q12841	15.00655777	3.907521182	Follistatin-related protein 1 OS=Homo sapiens OX=9606 GN=FSTL1 PE=1 SV=1
O00204	4.150719187	2.053361331	Sulfotransferase 2B1 OS=Homo sapiens OX=9606 GN=SULT2B1 PE=1 SV=2
Q9HAT2	3.433460287	1.779663277	Sialate O-acetylesterase OS=Homo sapiens OX=9606 GN=SIAE PE=1 SV=1
P05198	29.71591461	4.893163881	Eukaryotic translation initiation factor 2 subunit 1 OS=Homo sapiens OX=9606 GN=EIF2S1 PE=1 SV=3
P30711	3.041732501	1.604893284	Glutathione S-transferase theta-1 OS=Homo sapiens OX=9606 GN=GSTT1 PE=1 SV=4
Q9NV96	2.965886286	1.568463285	Cell cycle control protein 50A OS=Homo sapiens OX=9606 GN=TMEM30A PE=1 SV=1
Q8IWA5	43.50142449	5.442990739	Choline transporter-like protein 2 OS=Homo sapiens OX=9606 GN=SLC44A2 PE=1 SV=3
Q13093	3.762495423	1.911689827	Platelet-activating factor acetylhydrolase OS=Homo sapiens OX=9606 GN=PLA2G7 PE=1 SV=1
Q13510	13.599878	3.765521805	Acid ceramidase OS=Homo sapiens OX=9606 GN=ASAH1 PE=1 SV=5
O60701	4.504959753	2.171514217	UDP-glucose 6-dehydrogenase OS=Homo sapiens OX=9606 GN=UGDH PE=1 SV=1
Q15121	3.029635019	1.599144002	Astrocytic phosphoprotein PEA-15 OS=Homo sapiens OX=9606 GN=PEA15 PE=1 SV=2
P22692	7.115968981	2.831060222	Insulin-like growth factor-binding protein 4 OS=Homo sapiens OX=9606 GN=IGFBP4 PE=1 SV=2
Q9BRT3	3.14465557	1.652902009	Migration and invasion enhancer 1 OS=Homo sapiens OX=9606 GN=MIEN1 PE=1 SV=1
P60981	6.258368448	2.645786597	Destrin OS=Homo sapiens OX=9606 GN=DSTN PE=1 SV=3
P01210	4.23737355	2.083170317	Proenkephalin-A OS=Homo sapiens OX=9606 GN=PENK PE=1 SV=1
P0CG30	10.52747472	3.396087506	Glutathione S-transferase theta-2B OS=Homo sapiens OX=9606 GN=GSTT2B PE=1 SV=1

Q93099	5.633697615	2.49408213	Homogentisate 1,2-dioxygenase OS=Homo sapiens OX=9606 GN=HGD PE=1 SV=2
P14324	3.341301079	1.740409987	Farnesyl pyrophosphate synthase OS=Homo sapiens OX=9606 GN=FDPS PE=1 SV=4
Q96QK1	7.784569667	2.960617289	Vacuolar protein sorting-associated protein 35 OS=Homo sapiens OX=9606 GN=VPS35 PE=1 SV=2
Q5T440	5.304298122	2.407161863	Putative transferase CAF17, mitochondrial OS=Homo sapiens OX=9606 GN=IBA57 PE=1 SV=1
P0DOY2	11.30812452	3.499287769	Immunoglobulin lambda constant 2 OS=Homo sapiens OX=9606 GN=IGLC2 PE=1 SV=1
Q99988	10.5994811	3.405921734	Growth/differentiation factor 15 OS=Homo sapiens OX=9606 GN=GDF15 PE=1 SV=3
P19835	6.481906628	2.696418239	Bile salt-activated lipase OS=Homo sapiens OX=9606 GN=CEL PE=1 SV=3
P49902	4.091498111	2.032629186	Cytosolic purine 5'-nucleotidase OS=Homo sapiens OX=9606 GN=NT5C2 PE=1 SV=1
P09467	3.893391219	1.961027318	Fructose-1,6-bisphosphatase 1 OS=Homo sapiens OX=9606 GN=FBP1 PE=1 SV=5
Q6PCB0	12.3314598	3.624271691	von Willebrand factor A domain-containing protein 1 OS=Homo sapiens OX=9606 GN=VWA1 PE=1 SV=1
P98160	13.00822869	3.701352621	Basement membrane-specific heparan sulfate proteoglycan core protein OS=Homo sapiens OX=9606 GN=HSPG2 PE=1 SV=4
Q9NZZ3	4.312775896	2.108616751	Charged multivesicular body protein 5 OS=Homo sapiens OX=9606 GN=CHMP5 PE=1 SV=1
P98095	6.705881136	2.745426911	Fibulin-2 OS=Homo sapiens OX=9606 GN=FBLN2 PE=1 SV=2
Q53GD3	7.328894535	2.873595604	Choline transporter-like protein 4 OS=Homo sapiens OX=9606 GN=SLC44A4 PE=1 SV=2
P49588	5.328826754	2.41381793	Alanine--tRNA ligase, cytoplasmic OS=Homo sapiens OX=9606 GN=AARS1 PE=1 SV=2
Q9UBR2	5.087768742	2.347033096	Cathepsin Z OS=Homo sapiens OX=9606 GN=CTSZ PE=1 SV=1
P01859	13.00959161	3.70150377	Immunoglobulin heavy constant gamma 2 OS=Homo sapiens OX=9606 GN=IGHG2 PE=1 SV=2
Q9Y2S2	3.048663465	1.608176903	Lambda-crystallin homolog OS=Homo sapiens OX=9606 GN=CRYL1 PE=1 SV=3
O15296	14.11522327	3.819180044	Polyunsaturated fatty acid lipoxygenase ALOX15B OS=Homo sapiens OX=9606 GN=ALOX15B PE=1 SV=3
P21953	3.36758336	1.751713658	2-oxoisovalerate dehydrogenase subunit beta, mitochondrial OS=Homo sapiens OX=9606 GN=BCKDHB PE=1 SV=2
O43278	10.9858957	3.457580595	Kunitz-type protease inhibitor 1 OS=Homo sapiens OX=9606 GN=SPINT1 PE=1 SV=2
P19652	54.9672252	5.780499747	Alpha-1-acid glycoprotein 2 OS=Homo sapiens OX=9606 GN=ORM2 PE=1 SV=2
P62701	2.999936341	1.584931887	40S ribosomal protein S4, X isoform OS=Homo sapiens OX=9606 GN=RPS4X PE=1 SV=2
P20073	3.30778213	1.725864213	Annexin A7 OS=Homo sapiens OX=9606 GN=ANXA7 PE=1 SV=3
Q9UBG3	7.659518612	2.937253724	Cornulin OS=Homo sapiens OX=9606 GN=CRNN PE=1 SV=1
Q8WVQ1	4.644237166	2.215441648	Soluble calcium-activated nucleotidase 1 OS=Homo sapiens OX=9606 GN=CANT1 PE=1 SV=1
Q6UX06	32.9818773	5.043601613	Olfactomedin-4 OS=Homo sapiens OX=9606 GN=OLFM4 PE=1 SV=1
P30048	3.276824745	1.712298515	Thioredoxin-dependent peroxide reductase, mitochondrial OS=Homo sapiens OX=9606 GN=PRDX3 PE=1 SV=3

Q13557	3.499487877	1.80714381	Calcium/calmodulin-dependent protein kinase type II subunit delta OS=Homo sapiens OX=9606 GN=CAMK2D PE=1 SV=3
Q15436	30.97945328	4.953239779	Protein transport protein Sec23A OS=Homo sapiens OX=9606 GN=SEC23A PE=1 SV=2
Q8TB22	2.893260681	1.532696315	Spermatogenesis-associated protein 20 OS=Homo sapiens OX=9606 GN=SPATA20 PE=2 SV=3
Q14764	3.548511378	1.827213932	Major vault protein OS=Homo sapiens OX=9606 GN=MVP PE=1 SV=4
O00194	13.41183172	3.745434382	Ras-related protein Rab-27B OS=Homo sapiens OX=9606 GN=RAB27B PE=1 SV=4
Q09666	15.13570376	3.919883852	Neuroblast differentiation-associated protein AHNAK OS=Homo sapiens OX=9606 GN=AHNAK PE=1 SV=2
A4D263	4.494816138	2.168262103	Spermatogenesis-associated protein 48 OS=Homo sapiens OX=9606 GN=SPATA48 PE=2 SV=2
O15382	4.489139227	2.166438841	Branched-chain-amino-acid aminotransferase, mitochondrial OS=Homo sapiens OX=9606 GN=BCAT2 PE=1 SV=2
P62879	3.394632619	1.763255448	Guanine nucleotide-binding protein G(I)/G(S)/G(T) subunit beta-2 OS=Homo sapiens OX=9606 GN=GNB2 PE=1 SV=3
P14735	10.6675183	3.41515268	Insulin-degrading enzyme OS=Homo sapiens OX=9606 GN=IDE PE=1 SV=4
O95716	30.59534406	4.935240218	Ras-related protein Rab-3D OS=Homo sapiens OX=9606 GN=RAB3D PE=1 SV=1
Q8IYS1	10.21220797	3.352222919	Xaa-Arg dipeptidase OS=Homo sapiens OX=9606 GN=PM20D2 PE=1 SV=2
O00560	16.65170695	4.057598169	Syntenin-1 OS=Homo sapiens OX=9606 GN=SDCBP PE=1 SV=1
P54802	7.429014479	2.893170838	Alpha-N-acetylglucosaminidase OS=Homo sapiens OX=9606 GN=NAGLU PE=1 SV=2
Q687X5	5.244862043	2.390904826	Metalloreductase STEAP4 OS=Homo sapiens OX=9606 GN=STEAP4 PE=1 SV=1
P19801	5.19491046	2.377098883	Amiloride-sensitive amine oxidase [copper-containing] OS=Homo sapiens OX=9606 GN=AOC1 PE=1 SV=4
P51812	2.8447346	1.508294062	Ribosomal protein S6 kinase alpha-3 OS=Homo sapiens OX=9606 GN=RPS6KA3 PE=1 SV=1
O75351	5.776091563	2.530093612	Vacuolar protein sorting-associated protein 4B OS=Homo sapiens OX=9606 GN=VPS4B PE=1 SV=2
P53004	6.83739504	2.773446781	Biliverdin reductase A OS=Homo sapiens OX=9606 GN=BLVRA PE=1 SV=2
O43505	13.51315793	3.756292957	Beta-1,4-glucuronyltransferase 1 OS=Homo sapiens OX=9606 GN=B4GAT1 PE=1 SV=1
Q9BTY2	3.544086024	1.825413622	Plasma alpha-L-fucosidase OS=Homo sapiens OX=9606 GN=FUCA2 PE=1 SV=2
Q96DG6	6.391652919	2.676189069	Carboxymethylenebutenolidase homolog OS=Homo sapiens OX=9606 GN=CMBL PE=1 SV=1
Q08209	18.43520705	4.204391714	Serine/threonine-protein phosphatase 2B catalytic subunit alpha isoform OS=Homo sapiens OX=9606 GN=PPP3CA PE=1 SV=1
Q12805	4.352904066	2.121978225	EGF-containing fibulin-like extracellular matrix protein 1 OS=Homo sapiens OX=9606 GN=EFEMP1 PE=1 SV=2
P30740	12.28544885	3.618878663	Leukocyte elastase inhibitor OS=Homo sapiens OX=9606 GN=SERPINB1 PE=1 SV=1
Q15828	3.857297565	1.947590444	Cystatin-M OS=Homo sapiens OX=9606 GN=CST6 PE=1 SV=1
P54108	27.08851145	4.759609213	Cysteine-rich secretory protein 3 OS=Homo sapiens OX=9606 GN=CRISP3 PE=1 SV=1

Q14894	5.191376529	2.37611713	Ketimine reductase mu-crystallin OS=Homo sapiens OX=9606 GN=CRYM PE=1 SV=1
P28799	9.12895555	3.19044981	Progranulin OS=Homo sapiens OX=9606 GN=GRN PE=1 SV=2
O00462	7.343908688	2.876548121	Beta-mannosidase OS=Homo sapiens OX=9606 GN=MANBA PE=1 SV=3
Q13449	15.51815728	3.955885349	Limbic system-associated membrane protein OS=Homo sapiens OX=9606 GN=LSAMP PE=1 SV=2
P01876	18.4190225	4.203124595	Immunoglobulin heavy constant alpha 1 OS=Homo sapiens OX=9606 GN=IGHA1 PE=1 SV=2
O75629	4.05190433	2.018600111	Protein CREG1 OS=Homo sapiens OX=9606 GN=CREG1 PE=1 SV=1
P16035	9.97536112	3.31836907	Metalloproteinase inhibitor 2 OS=Homo sapiens OX=9606 GN=TIMP2 PE=1 SV=2
P21980	4.607613925	2.204019838	Protein-glutamine gamma-glutamyltransferase 2 OS=Homo sapiens OX=9606 GN=TGM2 PE=1 SV=2
P24593	17.78958109	4.152960633	Insulin-like growth factor-binding protein 5 OS=Homo sapiens OX=9606 GN=IGFBP5 PE=1 SV=1
Q9UBC9	105.8837686	6.726337638	Small proline-rich protein 3 OS=Homo sapiens OX=9606 GN=SPRR3 PE=1 SV=2

94 Down-regulated

ID	***FC***	***Log2FC***	***Description***
Q9NX62	0.312544829	-1.67786496	Golgi-resident adenosine 3',5'-bisphosphate 3'-phosphatase OS=Homo sapiens OX=9606 GN=BPNT2 PE=1 SV=1
P06753-3	0.008684693	-6.847309476	Isoform 3 of Tropomyosin alpha-3 chain OS=Homo sapiens OX=9606 GN=TPM3
Q9Y2B4	0.339713452	-1.557609747	TP53-target gene 5 protein OS=Homo sapiens OX=9606 GN=TP53TG5 PE=1 SV=1
P04554	0.152219141	-2.71577831	Protamine-2 OS=Homo sapiens OX=9606 GN=PRM2 PE=1 SV=3
Q5FVE4	0.23007597	-2.119817781	Long-chain-fatty-acid--CoA ligase ACSBG2 OS=Homo sapiens OX=9606 GN=ACSBG2 PE=1 SV=2
Q9NZ01	0.13286174	-2.912002386	Very-long-chain enoyl-CoA reductase OS=Homo sapiens OX=9606 GN=TECR PE=1 SV=1
Q9Y371	0.348256042	-1.521779713	Endophilin-B1 OS=Homo sapiens OX=9606 GN=SH3GLB1 PE=1 SV=1
Q8WY22	0.343946911	-1.539742197	BRI3-binding protein OS=Homo sapiens OX=9606 GN=BRI3BP PE=1 SV=1
P06748	0.182879832	-2.451032112	Nucleophosmin OS=Homo sapiens OX=9606 GN=NPM1 PE=1 SV=2
Q92743	0.265604891	-1.912646384	Serine protease HTRA1 OS=Homo sapiens OX=9606 GN=HTRA1 PE=1 SV=1
P22626	0.16076873	-2.636941272	Heterogeneous nuclear ribonucleoproteins A2/B1 OS=Homo sapiens OX=9606 GN=HNRNPA2B1 PE=1 SV=2
Q02218	0.207329237	-2.270004519	2-oxoglutarate dehydrogenase, mitochondrial OS=Homo sapiens OX=9606 GN=OGDH PE=1 SV=3
P68431	0.258196334	-1.953459577	Histone H3.1 OS=Homo sapiens OX=9606 GN=H3C1 PE=1 SV=2
P07910	0.183436466	-2.44664763	Heterogeneous nuclear ribonucleoproteins C1/C2 OS=Homo sapiens OX=9606 GN=HNRNPC PE=1 SV=4
P05023	0.179821717	-2.475360828	Sodium/potassium-transporting ATPase subunit alpha-1 OS=Homo sapiens OX=9606 GN=ATP1A1 PE=1 SV=1

O94973	0.26511271	-1.915322257	AP-2 complex subunit alpha-2 OS=Homo sapiens OX=9606 GN=AP2A2 PE=1 SV=2
Q9Y6M9	0.235391086	-2.086868407	NADH dehydrogenase [ubiquinone] 1 beta subcomplex subunit 9 OS=Homo sapiens OX=9606 GN=NDUFB9 PE=1 SV=3
P21810	0.193259558	-2.37138833	Biglycan OS=Homo sapiens OX=9606 GN=BGN PE=1 SV=2
P26583	0.317965676	-1.653057056	High mobility group protein B2 OS=Homo sapiens OX=9606 GN=HMGB2 PE=1 SV=2
P51854	0.253423544	-1.980377535	Transketolase-like protein 1 OS=Homo sapiens OX=9606 GN=TKTL1 PE=1 SV=2
Q16566	0.317714771	-1.654195929	Calcium/calmodulin-dependent protein kinase type IV OS=Homo sapiens OX=9606 GN=CAMK4 PE=1 SV=1
Q9H008	0.177790971	-2.491746033	Phospholysine phosphohistidine inorganic pyrophosphate phosphatase OS=Homo sapiens OX=9606 GN=LHPP PE=1 SV=2
Q92598	0.192691845	-2.375632575	Heat shock protein 105 kDa OS=Homo sapiens OX=9606 GN=HSPH1 PE=1 SV=1
Q9Y376	0.344068412	-1.539232648	Calcium-binding protein 39 OS=Homo sapiens OX=9606 GN=CAB39 PE=1 SV=1
O00253	0.243660983	-2.037052841	Agouti-related protein OS=Homo sapiens OX=9606 GN=AGRP PE=1 SV=1
P23435	0.308837342	-1.695080893	Cerebellin-1 OS=Homo sapiens OX=9606 GN=CBLN1 PE=1 SV=1
Q8IY17	0.068624161	-3.865139589	Patatin-like phospholipase domain-containing protein 6 OS=Homo sapiens OX=9606 GN=PNPLA6 PE=1 SV=3
Q9NP58	0.339933365	-1.556676122	ATP-binding cassette sub-family B member 6 OS=Homo sapiens OX=9606 GN=ABCB6 PE=1 SV=1
Q9Y365	0.331896072	-1.591196539	START domain-containing protein 10 OS=Homo sapiens OX=9606 GN=STARD10 PE=1 SV=2
Q8WWF5	0.230447521	-2.11748985	E3 ubiquitin-protein ligase ZNRF4 OS=Homo sapiens OX=9606 GN=ZNRF4 PE=1 SV=3
Q9Y2T7	0.132678464	-2.913993876	Y-box-binding protein 2 OS=Homo sapiens OX=9606 GN=YBX2 PE=1 SV=2
P12955	0.238530015	-2.067757281	Xaa-Pro dipeptidase OS=Homo sapiens OX=9606 GN=PEPD PE=1 SV=3
Q96T59	0.333887876	-1.582564386	CMT1A duplicated region transcript 15 protein OS=Homo sapiens OX=9606 GN=CDRT15 PE=2 SV=1
P62312	0.153733016	-2.701501062	U6 snRNA-associated Sm-like protein LSm6 OS=Homo sapiens OX=9606 GN=LSM6 PE=1 SV=1
P61158	0.211893168	-2.238591024	Actin-related protein 3 OS=Homo sapiens OX=9606 GN=ACTR3 PE=1 SV=3
Q9BRR6	0.163261829	-2.614740572	ADP-dependent glucokinase OS=Homo sapiens OX=9606 GN=ADPGK PE=1 SV=1
O75379	0.257100621	-1.959595003	Vesicle-associated membrane protein 4 OS=Homo sapiens OX=9606 GN=VAMP4 PE=1 SV=2
P35813	0.277709434	-1.848351906	Protein phosphatase 1A OS=Homo sapiens OX=9606 GN=PPM1A PE=1 SV=1
Q13765	0.288151614	-1.795099995	Nascent polypeptide-associated complex subunit alpha OS=Homo sapiens OX=9606 GN=NACA PE=1 SV=1
A0A1B0GUS4	0.226450062	-2.142735159	Ubiquitin-conjugating enzyme E2 L5 OS=Homo sapiens OX=9606 GN=UBE2L5 PE=2 SV=1
O00154	0.289557112	-1.788080163	Cytosolic acyl coenzyme A thioester hydrolase OS=Homo sapiens OX=9606 GN=ACOT7 PE=1 SV=3
Q9Y333	0.296040587	-1.756133111	U6 snRNA-associated Sm-like protein LSm2 OS=Homo sapiens OX=9606 GN=LSM2 PE=1 SV=1

Q6UXH1	0.273451646	-1.870642349	Protein disulfide isomerase CRELD2 OS=Homo sapiens OX=9606 GN=CRELD2 PE=1 SV=1
O95166	0.206507875	-2.275731294	Gamma-aminobutyric acid receptor-associated protein OS=Homo sapiens OX=9606 GN=GABARAP PE=1 SV=1
O75663	0.313308558	-1.674343918	TIP41-like protein OS=Homo sapiens OX=9606 GN=TIPRL PE=1 SV=2
O95373	0.330509371	-1.597236919	Importin-7 OS=Homo sapiens OX=9606 GN=IPO7 PE=1 SV=1
Q68CQ1	0.193249567	-2.37146291	Maestro heat-like repeat-containing protein family member 7 OS=Homo sapiens OX=9606 GN=MROH7 PE=2 SV=4
P08246	0.181863757	-2.459070036	Neutrophil elastase OS=Homo sapiens OX=9606 GN=ELANE PE=1 SV=1
O14672	0.342793616	-1.544587854	Disintegrin and metalloproteinase domain-containing protein 10 OS=Homo sapiens OX=9606 GN=ADAM10 PE=1 SV=1
Q9NRN7	0.312789448	-1.676736249	L-aminoadipate-semialdehyde dehydrogenase-phosphopantetheinyl transferase OS=Homo sapiens OX=9606 GN=AASDHPPT PE=1 SV=2
Q9C0E8	0.168138793	-2.572275469	Endoplasmic reticulum junction formation protein lunapark OS=Homo sapiens OX=9606 GN=LNPK PE=1 SV=2
P36405	0.220562761	-2.180738861	ADP-ribosylation factor-like protein 3 OS=Homo sapiens OX=9606 GN=ARL3 PE=1 SV=2
P07203	0.299367998	-1.740008086	Glutathione peroxidase 1 OS=Homo sapiens OX=9606 GN=GPX1 PE=1 SV=4
P24158	0.252249744	-1.987075289	Myeloblastin OS=Homo sapiens OX=9606 GN=PRTN3 PE=1 SV=3
P52272	0.217165915	-2.203130411	Heterogeneous nuclear ribonucleoprotein M OS=Homo sapiens OX=9606 GN=HNRNPM PE=1 SV=3
Q6UXG2	0.286548258	-1.803149969	Endosome/lysosome-associated apoptosis and autophagy regulator 1 OS=Homo sapiens OX=9606 GN=ELAPOR1 PE=1 SV=2
Q9NRY5	0.328162333	-1.60751844	Protein FAM114A2 OS=Homo sapiens OX=9606 GN=FAM114A2 PE=1 SV=4
P52888	0.317313451	-1.656019416	Thimet oligopeptidase OS=Homo sapiens OX=9606 GN=THOP1 PE=1 SV=2
Q8NFW8	0.289736527	-1.787186519	N-acylneuraminate cytidylyltransferase OS=Homo sapiens OX=9606 GN=CMAS PE=1 SV=2
P14543	0.179535287	-2.477660663	Nidogen-1 OS=Homo sapiens OX=9606 GN=NID1 PE=1 SV=3
P49321	0.281082072	-1.830936656	Nuclear autoantigenic sperm protein OS=Homo sapiens OX=9606 GN=NASP PE=1 SV=2
Q3SXM5	0.318436936	-1.65092041	Inactive hydroxysteroid dehydrogenase-like protein 1 OS=Homo sapiens OX=9606 GN=HSDL1 PE=1 SV=3
Q9BW27	0.29824529	-1.74542874	Nuclear pore complex protein Nup85 OS=Homo sapiens OX=9606 GN=NUP85 PE=1 SV=1
Q9GZY8	0.111382405	-3.166406743	Mitochondrial fission factor OS=Homo sapiens OX=9606 GN=MFF PE=1 SV=1
P08195	0.22933875	-2.124447955	4F2 cell-surface antigen heavy chain OS=Homo sapiens OX=9606 GN=SLC3A2 PE=1 SV=3
Q15833	0.181324702	-2.463352619	Syntaxin-binding protein 2 OS=Homo sapiens OX=9606 GN=STXBP2 PE=1 SV=2
P49458	0.333232178	-1.585400375	Signal recognition particle 9 kDa protein OS=Homo sapiens OX=9606 GN=SRP9 PE=1 SV=2
O95249	0.287069942	-1.800525816	Golgi SNAP receptor complex member 1 OS=Homo sapiens OX=9606 GN=GOSR1 PE=1 SV=1

Q9UHQ4	0.217018005	-2.204113355	B-cell receptor-associated protein 29 OS=Homo sapiens OX=9606 GN=BCAP29 PE=1 SV=2
Q3ZCQ8	0.278007984	-1.846801777	Mitochondrial import inner membrane translocase subunit TIM50 OS=Homo sapiens OX=9606 GN=TIMM50 PE=1 SV=2
P49788	0.041857068	-4.578384944	Retinoic acid receptor responder protein 1 OS=Homo sapiens OX=9606 GN=RARRES1 PE=1 SV=2
P35658	0.287120204	-1.80027324	Nuclear pore complex protein Nup214 OS=Homo sapiens OX=9606 GN=NUP214 PE=1 SV=2
O00233	0.166123193	-2.589674588	26S proteasome non-ATPase regulatory subunit 9 OS=Homo sapiens OX=9606 GN=PSMD9 PE=1 SV=3
O95825	0.295823838	-1.757189785	Quinone oxidoreductase-like protein 1 OS=Homo sapiens OX=9606 GN=CRYZL1 PE=1 SV=2
O95139	0.338535021	-1.562623007	NADH dehydrogenase [ubiquinone] 1 beta subcomplex subunit 6 OS=Homo sapiens OX=9606 GN=NDUFB6 PE=1 SV=3
Q13617	0.283435851	-1.818905841	Cullin-2 OS=Homo sapiens OX=9606 GN=CUL2 PE=1 SV=2
Q9UMQ6	0.314490154	-1.668913247	Calpain-11 OS=Homo sapiens OX=9606 GN=CAPN11 PE=2 SV=2
Q8WU17	0.276027098	-1.85711819	E3 ubiquitin-protein ligase RNF139 OS=Homo sapiens OX=9606 GN=RNF139 PE=1 SV=1
P62314	0.330799158	-1.595972532	Small nuclear ribonucleoprotein Sm D1 OS=Homo sapiens OX=9606 GN=SNRPD1 PE=1 SV=1
Q15831	0.305930143	-1.708725831	Serine/threonine-protein kinase STK11 OS=Homo sapiens OX=9606 GN=STK11 PE=1 SV=1
O95456	0.162570004	-2.620867005	Proteasome assembly chaperone 1 OS=Homo sapiens OX=9606 GN=PSMG1 PE=1 SV=1
P51991	0.178104302	-2.489205734	Heterogeneous nuclear ribonucleoprotein A3 OS=Homo sapiens OX=9606 GN=HNRNPA3 PE=1 SV=2
Q8WUM0	0.330657028	-1.596592527	Nuclear pore complex protein Nup133 OS=Homo sapiens OX=9606 GN=NUP133 PE=1 SV=2
Q8WVM8	0.244159642	-2.034103341	Sec1 family domain-containing protein 1 OS=Homo sapiens OX=9606 GN=SCFD1 PE=1 SV=4
P43243	0.057095276	-4.13048481	Matrin-3 OS=Homo sapiens OX=9606 GN=MATR3 PE=1 SV=2
Q6XQN6	0.186482662	-2.422886593	Nicotinate phosphoribosyltransferase OS=Homo sapiens OX=9606 GN=NAPRT PE=1 SV=2
O00429	0.139644962	-2.840164571	Dynamin-1-like protein OS=Homo sapiens OX=9606 GN=DNM1L PE=1 SV=2
Q7Z3C6	0.326072811	-1.616733947	Autophagy-related protein 9A OS=Homo sapiens OX=9606 GN=ATG9A PE=1 SV=3
Q9NRD1	0.10901228	-3.197437433	F-box only protein 6 OS=Homo sapiens OX=9606 GN=FBXO6 PE=1 SV=1
Q03169	0.209342505	-2.256062827	Tumor necrosis factor alpha-induced protein 2 OS=Homo sapiens OX=9606 GN=TNFAIP2 PE=1 SV=2
P62263	0.291832181	-1.776789112	40S ribosomal protein S14 OS=Homo sapiens OX=9606 GN=RPS14 PE=1 SV=3
P00751	0.245013756	-2.029065348	Complement factor B OS=Homo sapiens OX=9606 GN=CFB PE=1 SV=2
P50454	0.164356377	-2.605100658	Serpin H1 OS=Homo sapiens OX=9606 GN=SERPINH1 PE=1 SV=2
O75955	0.224899228	-2.152649385	Flotillin-1 OS=Homo sapiens OX=9606 GN=FLOT1 PE=1 SV=3
