# Supplementary figures and images for: Molecular Remodeling of the Sperm Proteome Following Varicocele Sclero-Embolization: Implications for Semen Quality Improvement
Source: Proteomes. 2025 Jul 15;13(3):34. doi: 10.3390/proteomes13030034 (PMC12286009; doi:10.3390/proteomes13030034)

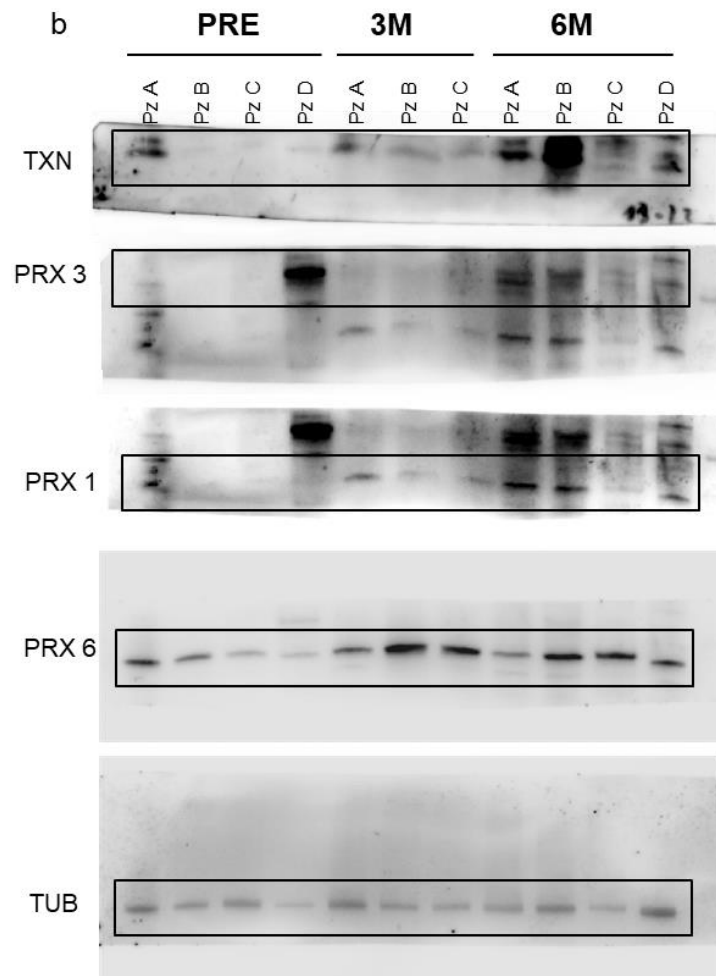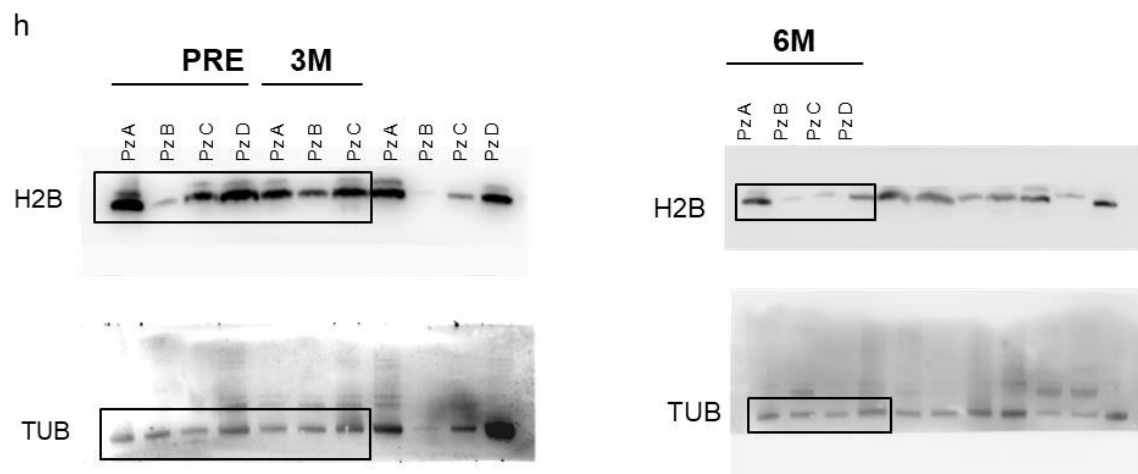

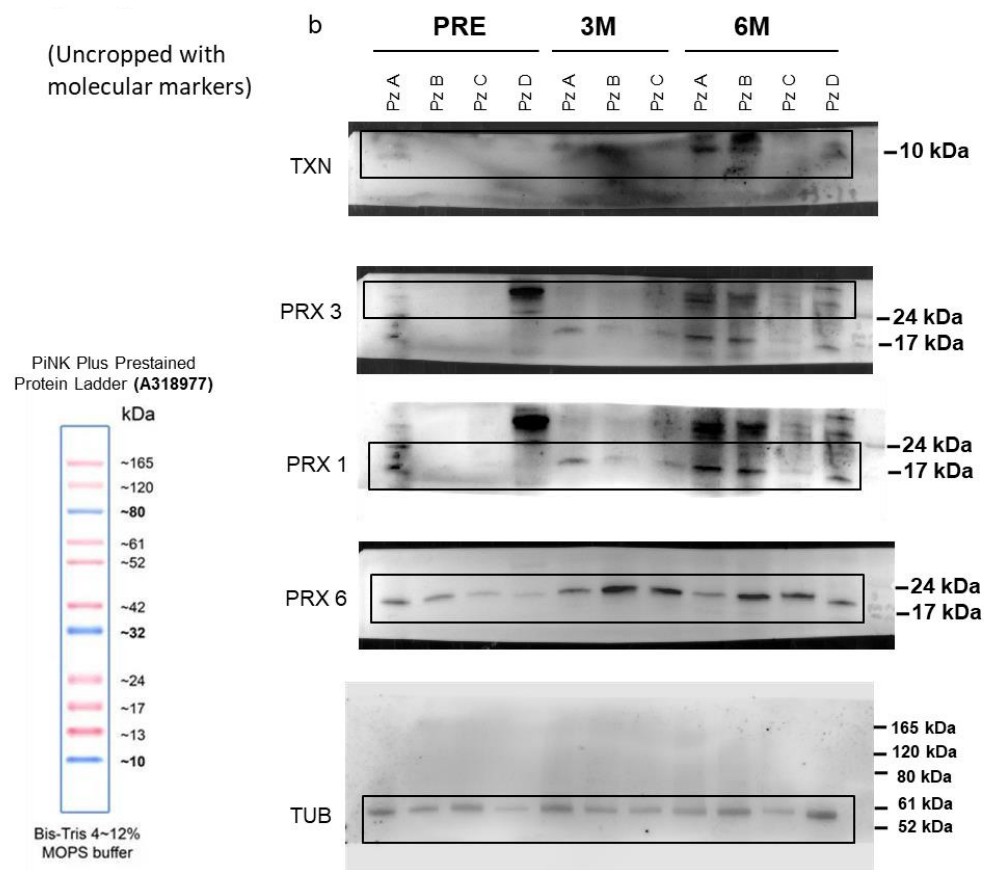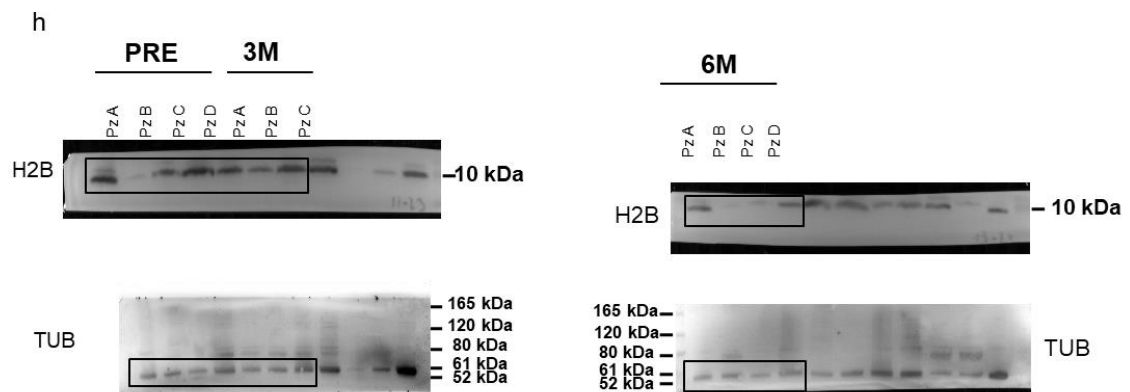

Supplement: Supplementary file 1 [file proteomes-13-00034-s001.zip › original blots.pdf]
